# Exploring Innovative Solutions for Quality of Life and Care of Bed-Ridden Nursing Home Residents through Codesign Sessions

**DOI:** 10.1155/2015/185054

**Published:** 2015-10-12

**Authors:** J. van Hoof, M. H. Wetzels, A. M. C. Dooremalen, R. A. Overdiep, M. E. Nieboer, A. M. E. Eyck, P. J. L. M. van Gorkom, E. L. M. Zwerts-Verhelst, S. Aarts, C. Vissers-Luijcks, C. S. van der Voort, M. J. G. A. Moonen, H. A. van de Vrande, C. J. M. L. van Dijck-Heinen, E. J. M. Wouters

**Affiliations:** ^1^Fontys University of Applied Sciences, Fontys EGT-Centre for Healthcare and Technology, Dominee Theodor Fliednerstraat 2, 5631 BN Eindhoven, Netherlands; ^2^Fontys University of Applied Sciences, Institute of Allied Health Professions, Dominee Theodor Fliednerstraat 2, 5631 BN Eindhoven, Netherlands; ^3^Department of Industrial Design, Eindhoven University of Technology, Den Dolech 2, 5612 AZ Eindhoven, Netherlands; ^4^Fontys School of People and Health Studies, Fontys University of Applied Sciences, Dominee Theodor Fliednerstraat 2, 5631 BN Eindhoven, Netherlands; ^5^Fontys University of Applied Sciences, Institute of Information and Communication Technology, Rachelsmolen 1, 5612 MA Eindhoven, Netherlands; ^6^Fontys University of Applied Sciences, Institute of Human Resource Management and Psychology, Emmasingel 28, 5611 AZ Eindhoven, Netherlands; ^7^Fontys University of Applied Sciences, Institute of Engineering, Rachelsmolen 1, 5612 MA Eindhoven, Netherlands

## Abstract

Bed-ridden nursing home residents are in need of environments which are homelike and facilitate the provision of care. Design guidance for this group of older people is limited. This study concerned the exploration and generation of innovative environmental enrichment scenarios for bed-ridden residents. This exploration was conducted through a combination of participatory action research with user-centred design involving 56 professional stakeholders in interactive work sessions. This study identified numerous design solutions, both concepts and products that are available on the marketplace and that on a higher level relate to improvements in resident autonomy and the supply of technological items and architectural features. The methodology chosen can be used to explore the creative potential of stakeholders from the domain of healthcare in product innovation.

## 1. Introduction

There are approximately 165,000 persons residing in Dutch long-term care institutions [1]. This is equivalent to around 6% of the Dutch population aged over 65. Nursing homes are often the final place of residence for the occupants. It is, therefore, important that the nursing home is not only a place where care is delivered, but also a place where one lives and feels truly at home making use of a wide array of architectural and technological solutions [1, 2]. This also applies to nursing home residents who are bed-ridden due to their declining health and physical fitness. Bed-ridden residents are confined to bed because of illness or infirmity, especially for a long or indefinite period. This group faces many difficulties in terms of activities of daily living and mobility. The percentage of nursing home residents who are permanently or sometimes bed-ridden was estimated by the Netherlands Institute for Social Research to be about 25%, with the highest percentages found in psychogeriatric wards [3].

To date, not much research has been done on people who are bed-ridden or staying in bed for prolonged periods of time, both in long-term care and hospitals, and how they perceive the space around them. Annemans et al. [4] cited the work by van der Geest and Mommersteeg [5], who concluded that the role of the bed in the general hospital experience is largely under researched: van der Geest and Mommersteeg [5, pages 11-12],* “[t]he importance of the bed for health is obvious [, and] beds in hospitals and other medical settings have a special relation to health. They are tools to organise and realise health recovery. [… Hospital] beds are not meant to provide privacy but rather to allow for optimal access to the patient by clinical staff. Hospital beds are surrounded by medical care providers and medical equipment, facilitating permanent surveillance and the possibility of intervention.*” In addition, Annemans et al. [4] showed that persons who are bed-ridden, including patients in hospitals, have a different experience and perception of the space around them. For instance, while being transported through the hospital, the bed is an important mediator between the patient and his or her environment. Annemans et al. [4] concluded that most research on beds and transport seems often restricted to functional matters such as organisation and ergonomics.

The situation in well-facilitated nursing homes, in terms of design and technology, is assumed to be similar to that of hospitals. There are, however, significant differences. As mentioned before, a nursing home is a place of residence, and many residents deal with the consequences of dementia syndrome affecting one's perception and abilities, resulting in further limitations to one's resilience and autonomy [6]. Given the transitions in Dutch long-term care, it is expected that the number of bed-ridden nursing home residents, both with a somatic and a psychogeriatric background will increase. This is a direct consequence of the increased threshold for admission, which is related to one's health status and the ability to carry out activities of daily living independently or with the help of home care services [1]. The way long-term care settings are designed for bed-ridden nursing home residents is different from that for residents who have a degree of mobility. This is because the space bed-ridden residents are able to access and use independently is so limited. This should have implications to the character and appearance of buildings. Apart from guidance concerning fire safety that exists on a national level [7], there is practically no design guidance for this group of residents.

The design and construction of nursing homes are a complex and dynamic process, as the very design of these buildings forces us to consider the technology-human interface directly in terms of living-space, ethics, and social priorities [8]. Moreover, the design process includes a large variety of stakeholders, who further add to the complexity. Technology and architectural solutions can support the well-being, activities of daily living, and quality of life of older residents and support and optimize the work processes of healthcare professionals and professionals in the domain of maintenance [9–11]. In order to meet the needs of future nursing home residents, in particular those who are bed-ridden, new creative and inclusive approaches are needed to come up with new and innovative design solutions. In addition, Annemans et al. [4] mentioned that spatial aspects, for instance, that of the direct environment of a bed-ridden resident, cannot be studied isolated from the activities going on in the spaces considered. Depending on what is happening and who is involved, each experience is shaped differently.

The aim of this study was to explore and generate design concepts for the direct environment of bed-ridden residents of nursing homes through interdisciplinary participatory design sessions. These design concepts should consist of innovative environmental enrichment scenarios, which, in this study, concern the enhancement of a person's physical or social environment.

## 2. Methodology

### 2.1. Type of Research Methodology: Participatory Action Research and User-Centred Design

Delhoofen [12] stated that, in the concept phase of a design process, also called the creative phase, creativity is allowed. Creativity, on the one hand, is an important source of inspiration for any dynamic organization, and, on the other hand, it is a capricious and uncontrollable phenomenon which defies any formalization and set of rules. Creativity is hard to manage, even though this is often necessary to find new solutions for complex problems. According to Delhoofen [12], creativity is the ability to ignore restrictive frameworks of thinking and to find original solutions for both everyday and new problems. Creativity is not the same as innovation, but creativity is necessary to come to new innovations. We define innovation as the creation of new products and the improvement and differentiation of products.

A qualitative methodology called participatory action research was used for this study and it provides a tool to deal with the complexity of the design challenge and stakeholder-related needs [13–18]. According to Madden et al. [15], participative frameworks have been shown to be useful in design of environments, and participatory action research has a long history of use with disadvantaged groups in order to assist them to improve their living conditions, with researchers and the research subjects working collaboratively to develop solutions for community issues. Moreover, participatory action research “*involves practitioners as both subjects and co-researchers*” [13, page 613]. According to Howard and Somerville [17], participatory action research is a common methodology among designers but is not often framed as a codesign practice. In this study, participatory action research is integrated with activities of making [19], such as scenario writing and storyboarding, performance ethnography, and collaborative design and prototyping [20–22]. According to Sanders and Stappers [19], “making” is an important activity for designers, as one can bring insights to the surface. Prototypes can play a number of roles, such as evoking a focused discussion in a team, the testing of a hypothesis, and confronting others as the theory is not hidden in abstraction. Other types of visualisation such as scenarios and storyboards can be made to allow designers to experience, test, transform, develop, and complete early ideas [19]. Sanders and Stappers [19, page 6] stated that “*in the very earliest phase of the design process, the focus is on using making activities for making sense of the future. Here, making activities are used as vehicles for collectively (e.g. designers and codesigners together) exploring, expressing and testing hypotheses about future ways of living.*” This is why the current study focuses on scenarios and prototyping as a way to think about the future of nursing home care for bed-ridden residents. According to the map of design research by Sanders and Stappers [19, 23], the current research can be classified as user-centred design, more than participatory design research, as end users (bed-ridden nursing home residents) are not included in the study but seen as the subject. At the same time, care professionals, who are end-users of the solutions identified in this study, are also stakeholders who benefit from these solutions. Therefore, we do qualify this study as participatory design research.

### 2.2. Location, Researchers, and Participants

**Figure 1:**
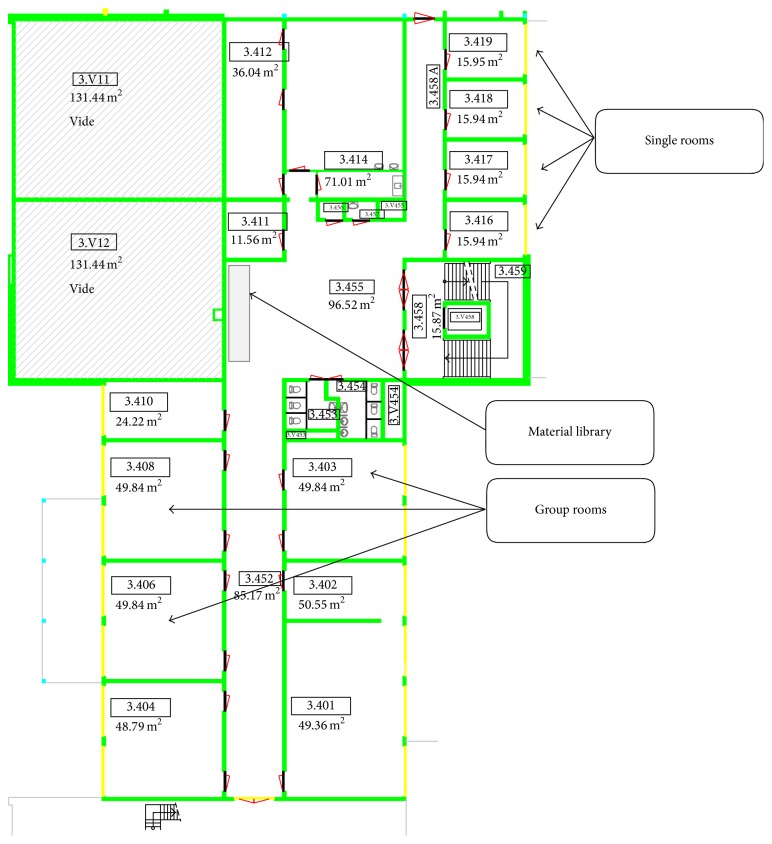
Layout of the rooms at the Fontys School of People and Health Studies, Eindhoven, in which the sessions took place.

**Figure 2:**
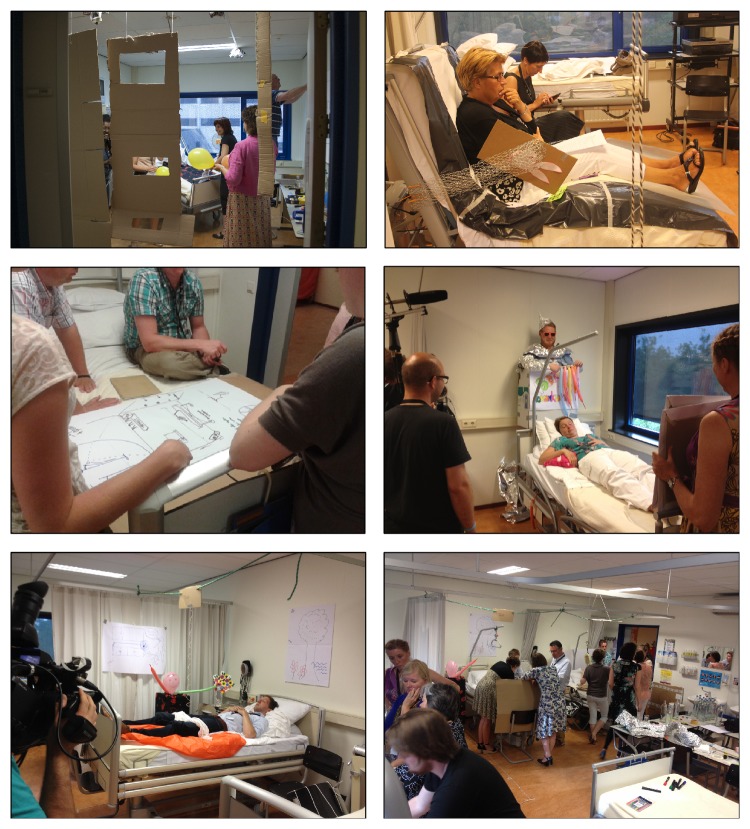
Working together to generate ideas, followed by the enactment and filming of the scenarios.

Work sessions were held in the evening of 18 June 2013 at the educational hospital wards of the Fontys School of People and Health Studies, Eindhoven, the Netherlands (Figure 1). The large nursing rooms (containing 4 beds) were about 50 m^2^ and the small rooms (containing 1 bed) 16 m^2^ in size. Each session lasted for 120–150 minutes. A total of 12 scenarios were made by 56 participants (Table 1, Figure 2) during the design improvisation sessions that were guided by creative facilitators (10 professional group facilitators and 9 assistant with a background in industrial design and creative techniques). All session facilitators and assistants had been briefed about the procedures and were provided with a manual and an instruction guide prior to the start of the sessions. The role for the session facilitator was to stimulate creativity, supporting the creative process, and to obtain a maximum of variety in responses and input. In the group rooms, session facilitators and assistants supported the various groups of participants. Apart from these facilitators and assistants, JVH, RO, and AD managed the overall processes of the sessions, supported the supply of materials, and collected written documentation. The participants of this study had a mixed background in healthcare, design, housing, and technology (Table 1).

### 2.3. Procedure and Assignment

At the start of each collaborative design session, a session facilitator welcomed the participants and explained the procedure. Informed consent, covering the permission to use of all written, videographic, and photographic materials, was obtained from the participants at the start of the session. The participants were invited to consider the beds in the rooms as actual nursing home beds and to enhance the environment with materials presented to them in a shared “material library,” including paper and card board, sticky tape and ropes, balloons, plastic covers, and various other do-it-yourself materials. These materials allowed the participants with a high degree of freedom to shape the modifications to the bed and use their imagination. The participants were told that the new scenarios and concepts mattered, not the level of perfection of the results or the execution of the modifications. The 12 scenarios that the participants developed were based on a persona (a fictional character) of a nursing home resident [21, 22, 24, 25]. Triantafyllakos et al. [21] stated that fictional characters have been used as user representatives, either substituting actual users or supporting idea generation. Their foremost objective is to facilitate the identification of user needs and goals and to support the development of detailed and comprehensive scenarios [21]. The interdisciplinary approach to the sessions was used to minimize stereotypical thinking [26]. Context scenarios were defined as a short story, which represented the use of the bed environment by the resident. The scenarios were represented by making actual changes and adaptations to the beds and the direct surroundings using a wide range of materials, which symbolized actual environmental modifications (Figure 2). At the end of the session, the personas were enacted by participants from each group who served as proxy residents (Figure 2). As creativity cannot be forced upon participants, it was important to create optimal conditions in which new ideas can come to the fore. For creativity to thrive, participants had to be comfortable, motivated, and stimulated through challenging goals [12]. Triantafyllakos et al. [21] found that design alter egos technique liberated the majority of participants from the fear of straightforwardly exposing themselves, supported and enhanced their introspection, stimulated their creativity, and helped to establish an informal and constructive atmosphere throughout the design sessions.

### 2.4. Data Analysis

The scenarios of the sessions were each documented on paper by the participants and filmed by a professional camera crew for further analysis. This is in line with the narrative videotaping approach to “storytelling laboratory” described by Bate and Robert [22]. The results of all 12 scenarios and the rationale behind each one of them (combination of a context scenario and requirements analysis) are presented as case studies containing potential design innovations that can be thought-through and developed in more detail for implementation in the nursing home setting. The transcripts of the scenarios were analysed in conjunction with the videos, in order to also include visual data. The data were analysed based on the six phases by Braun and Clarke [27] and in conjunction with the steps of content analysis by Krippendorff [28].

First, the transcripts were each read in their entirety. Then, they were read a second time to develop open codes identifying design solutions. Open coding concerns the process of unravelling all of the collected data into fragments or codes. Thereafter, codes were added to the transcripts. Similar codes and quotes were clustered and labelled, and themes emerged from this process. Together, the research team organised the codes and clustered them into smaller thematic groups. The final themes were grouped in amalgamation with graphical material from the films that reflected aspects of these themes, which is a form of axial coding. Thereafter, these themes were reviewed and then defined and named. These themes are made up of design concepts and solutions, which in turn could also be clustered in major themes, that is, to which level of human functioning they relate. Researcher triangulation was applied during the entire process; for example, separate analyses of the scenarios were conducted by two of the authors (J. van Hoof, A. M. C. Dooremalen). This guarantees that the data are interpreted independently and from different perspectives. A method triangulation was applied due to the use of both written scenarios and video images.

## 3. Results

**Figure 3:**
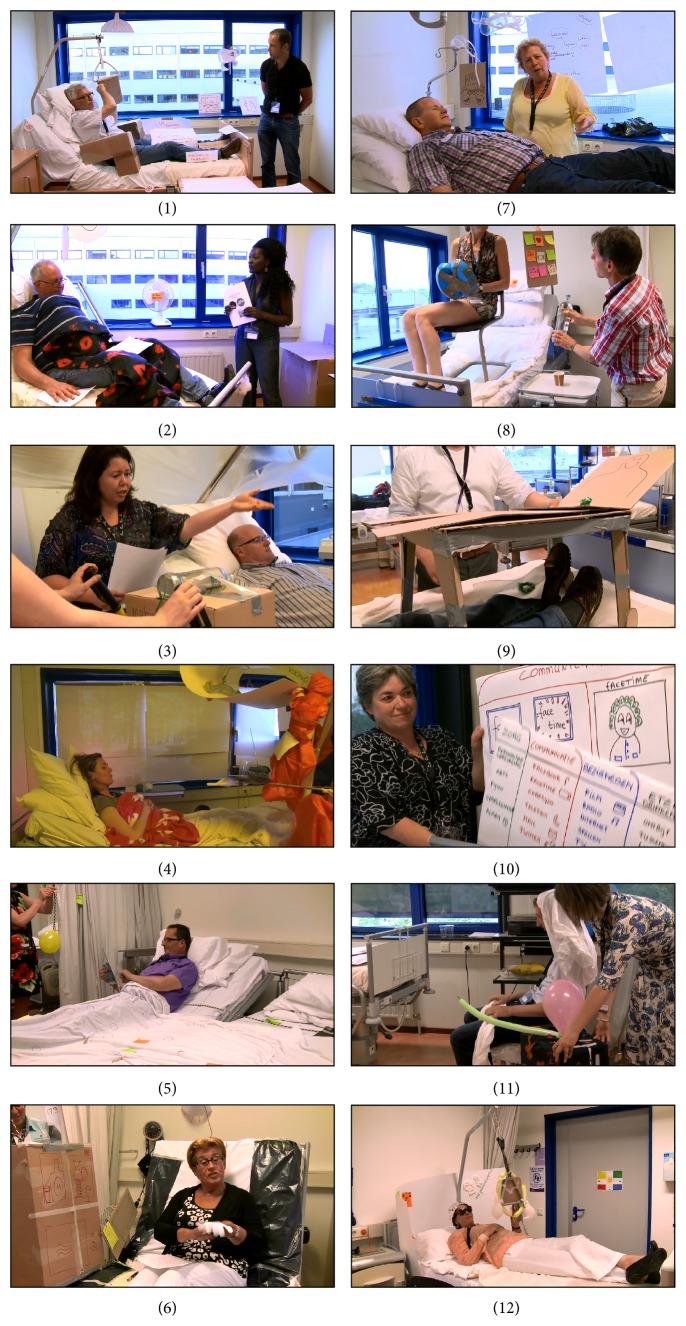
A composition of the twelve scenarios written and enacted by the participants.

The 12 scenarios contained suggestions that can be used for improving the living situation of bed-ridden nursing home residents or for changing product design and are given in the Appendix.  Figure 3 shows a composition of the enactments of each of these scenarios. The main themes which emerged from the analysis of the written and video materials are shown in Table 2. A high degree of convergence and data saturation has been found. The interdisciplinary character of the groups (and the interaction between participants) and the mixed location in both single-person and group rooms contributed to the richness of the scenarios and the data. Some of the themes found in this study are overarching (such as the use of tablet computers), whereas smaller themes are an elaboration of the functionalities one should use the tablet computer for (for instance, opening curtains).

The most frequently mentioned theme (11 out of 12) is the use of virtual and interactive walls for displaying pictures, television programs, videos, and materials from the past. This theme is followed by the possibility to have online contacts and to chat with relatives, friends, and fellow residents (8 out of 12). Seven out of twelve scenarios deal with the use of touch screens and tablet computers for the control of the space around them or to place orders for meals and plan the care tasks throughout the day and to control the environment such as curtains. Lights and luminaires, sounds, and music are mentioned (7 out of 12) as important areas for development when designing nursing home environments, for creating a proper atmosphere, for functionality, in order to stimulate day and night rhythms, and in order to make people feel more at ease through music and sounds of nature. The so-called transformer beds, beds that can change their shape into a chair, wheelchair, or bathtub are mentioned by 7 out of 12 scenarios and reflect new products on the market place: beds which can bend and turn and become like real chairs in which residents/patients can sit upright. These developments go together with the desire for smart mattress and smart beds measuring vital signs (6 out of 12) and special mattresses that improve comfort and prevent decubitus (6 out of 12). Seven out of twelve scenarios give attention to the free choice of food and drinks and the need for minibars and coffee corners where visitors can get something to drink. This should contribute to a sense of home and coziness.

Autonomy is an overarching or major theme, which encompasses the freedom and being in control of residents, which is reflected in the choice for meals, planning care activities, being in control of people entering the room, and so on. The smaller themes constituting this overarching theme are numerous. Robotics is a theme mentioned by 3 out of 12 scenarios, mainly in relation to transfers and delivering meals. Homelikeness and atmosphere are another overarching theme, of which smaller themes as flowers, furniture for relatives, and picture frames could be an elaboration.

## 4. Discussion and Conclusions

### 4.1. Reflection on Identified Design Solutions

The twelve scenarios resulting from this study contained numerous design ideas and solutions that can be used for improving the quality of life for bed-ridden nursing home residents. In this round of workshops, there was a lot of focus on technologies improving the autonomy of residents and for improving communication capabilities. Overall, the minor and major themes can be on different levels. For instance, tablet computers are things or items and are named as design solutions, whereas the autonomy relates to a personal experience or a human state. The computer tablets, however, can be used for achieving autonomy. The definition of environmental enrichment used in this study, which is the enhancement of a person's physical or social environment, allows for themes that relate to different levels. Various technological solutions were identified, which, apart from the aforementioned tablet computers and touchscreens, include assistive and medical technology such as special beds containing sensors, architectural modifications as windows, and building services solutions including heating, ventilation, and air-conditioning systems and smart home technologies. Although some of the design solutions proposed are futuristic, the vast majority is already available on the marketplace. These innovations are not yet implemented on a large scale in practice, and, therefore, they are still innovative.

Concept generation was at the centre of the study. The goal was to come up with innovative and conceptual design concepts which can be developed into implementable solutions. This study welcomed five types of innovation as distinguished by Delhoofen [12], namely, new products, product improvements, product differentiation, service innovations, and process innovations. The most important aspects of improvement to be achieved with innovations are savings, improvements of functionalities, and extensions of functionalities [12]. Whether the identified solutions can in fact contribute to improvements to healthcare is beyond the scope of the current work.

### 4.2. Applicability of the Methodology

The chosen approach to this study, participatory action research, has been used within the domain of public health care before. Its combination with user-centred design approaches, however, is relatively new within the domain of healthcare. A small number of scientific publications applying these methodologies within the domain of healthcare and technology are being published (for instance, [29, 30]). The current method had been shown successful in identifying aspects of healthcare facilities which are necessary for the quality of care and life of bed-ridden nursing home residents and for obtaining convergence in design solutions. The creative methodology is very suitable for bringing together people with different backgrounds and quality of knowledge. Cooperation in healthcare innovation is not uncommon in practice. Participatory action research aims to obtain “*knowledge and action directly useful to people, and also to empower people through the process of constructing and using their own knowledge*” [31, p. 225]. The methodology could, therefore, also be applied within healthcare settings in order to think about improving products, environments, and services and, most of all, to involve nondesigning professionals in a design process. In this methodology, all participants have a role to play, whether it is a designer or engineer or a healthcare specialist. It is, therefore, a truly inclusive design process, but inclusive design in this sense does not pertain to inclusive design or design-for-all. In addition, the participants working in commerce can use the methodology to improve products being designed and produced within their own enterprises. The methodology can also be used for educational practices, for instance, in multi- or interdisciplinary classes [32, 33], for a large spectrum of design challenges, as is currently being done.

### 4.3. Reflection on the Methodology of the Study

In this study, older persons, nursing home residents, and actual bed-ridden persons were not included in the creative sessions. This means that any solution that comes to the fore does not necessarily reflect the actual needs of older, bed-ridden people. At the same time, the participating healthcare professionals could provide a foundation for the design solutions based on their practical knowledge. In future sessions, one could think of including informal or family carers or professional actors trained in playing nursing home residents, in order to obtain more accurate scenarios. The current cohort of participants, as was discussed in the methodology section, could have based their scenarios on stereotypes of bed-ridden residents or could have portrayed them as somewhat active and independently functioning older people, which does not match with the actual health status and abilities of bed-ridden nursing home residents. Moreover, the methodology chosen may lead to scenarios that are enacted in too playful a manner.

Another potential weakness of the study is that participants are not aware of all innovations in the field, of those that appear on the market or are being researched in research and development (R&D) facilities. This may give a skewed perspective on the scenarios, which are either too optimistic or somewhat out-of-date. In the current study, scenarios are enacted using simple, low fidelity prototypes. This also means that concepts are not being developed into real innovative products, which can, in turn, be tested in the nursing home. This means that no field-evidence is being gathered, although the scenarios themselves are based on practice-based evidence. The evidence-based and performance-based designs (van Hoof et al. [34]) are becoming important tools in optimizing the spatial layout and interior design of healthcare facilities. Marquardt and Motzek [35, page 116] stated that “*because architects and designers are often not trained in research methods, they may feel somewhat overwhelmed by the terms used in the description of the methodology of scientific papers. Therefore, when designing healthcare facilities, they may not feel confident discussing the evidence available from research studies with members of the medical professions, who often display a profound knowledge of research methods.*” When evidence of certain measures is absent, or when new innovations need to be designed, a qualitative approach based on performance ethnography and collaborative design may be a good step in exchanging ideas and come up with new innovative design solutions. The duration of an optimal session should be researched. In the current study, the 2–2.5-hour period was considered to be sufficient for writing the scenario, building the scene, and enacting the scenario.

### 4.4. Conclusive Remarks

In conclusion, the chosen methodology may help practitioners identify potential solutions that exist to support the challenges in nursing home care. Moreover, the results of this study can be used to adequately design the private rooms and bed environments of nursing home residents in accordance with personal preferences and needs and to stimulate innovations in product design which may become available on the marketplace in the future.

## Figures and Tables

**Table 1:** List of sessions and the characteristics of the participants.

Group/scenario	Group facilitators and assistants	Room	Characteristics of participants	Background
1	CVL	3.416 (single-resident room)	*n* = 4 (1 female, 3 males)	Healthcare real estate, construction and architecture, and environmental health

2	SA, TW	3.417 (single-resident room)	*n* = 4 (3 females, 1 male)	Healthcare, interior design/architecture, and medical beds

3	LZV, LV	3.418 (single-resident room)	*n* = 5 (2 females, 3 males)	Interior design, healthcare, mobility aids, and electrical engineering

4	PG, CH	3.419 (single-resident room)	*n* = 5 (2 females, 3 male)	Healthcare technology, electrical engineering, ICT, and interactive technology

5	EW, JW	3.406 (group room)	*n* = 6 (4 females, 2 males)	Architecture, building physics, healthcare, and medical devices

6	MM, BP	3.406 (group room)	*n* = 6 (3 females, 3 males)	Healthcare, electrical engineering, healthcare technologies, and construction

7			*n* = 4 (1 female, 3 males)	Healthcare, electrical engineering
8	MN, NP, LOW, and CDH	3.403 (group room)	*n* = 5 (1 female, 4 males)	ICT, real estate, healthcare, and electrical engineering
9			*n* = 4 (1 female, 3 males)	Acoustics, assistive technologies, and healthcare

10			*n* = 4 (3 females, 1 male)	ICT, cleaning, healthcare, and municipality
11	CV, AE, TR, and MW	3.408 (group room)	*n* = 4 (2 females, 2 males)	Social innovation, assistive technologies, and technology innovation services
12			*n* = 5 (3 females, 2 males)	Building services, healthcare, electrical engineering, and acoustics

	Total: 10 facilitators, 9 assistants		*n* = 56 (26 females, 30 males)	

**Table 2:** Themes or design concepts emerging from the scenarios.

Themes	Scenario
Touchscreens, control panels, and tablet computers	1, 2, 5, 6, 8, 11, and 12
Transformer beds (into chairs and baths)	1, 3, 4, 6, 8, 9, and 12
Sensors in bed for measuring vital functions and analysis	1, 2, 3, 7, and 9
Massage mattress of modified mattress (decubitus)	1, 2, 3, 5, and 9
Arm rests	1
Virtual, interactive walls (displaying pictures, films), and picture frames	1, 2, 3, 4, 5, 6, 7, 8, 9, 11, and 12
Home wall with personal belongings	1
Low sill window	1
Sensors responding to movement of arms and legs for control	1, 6
Odour box	1, 7
Mobile furniture for relatives	1, 2, and 12
Workplace for relatives with ICT and Wi-Fi	1, 2
Moveable walls (privacy)	2
Flowers and plants	2, 3, and 5
Food and drinks services (for relatives)	2, 4, 5, 6, 8, 10, and 11
Devices and screens for online contacts (voice calls), chatting	2, 5, 6, 8, 9, 10, 11, and 12
Own choice for interior design	2
Wake-up call and good morning service	2, 4, 6
Atmosphere and homelikeness	2, 5, 7, 8, and 12
Moving experience ceiling	3
Lights, luminaires, sounds, and music	3, 4, 7, 8, 9, 11, and 12
Control buddy	3, 9
Mobile shower	3
Two-person bed	3, 5
Medical equipment unobtrusive	3
Robotics	4, 10, and 11
Smart window	4
Operable curtains	5, 6
Smart wardrobe	5
Doorbells, entrance control, and do-not-disturb button	5, 6, and 8
Mobile washbasin	5
Skylight	5
Mattress with temperature control/HVAC	5, 12
Bionic arms	6
Self-repetitive videos for restlessness	7
Wind machine	7
3D projector	7
Technology for sexual support	9
Planning assistant	10, 11
Control of technology based on eye movement	10
Need for face-to-face contact	11
Freedom to live where you want/mobile environment	12
Information glasses	12
Smart hat: control device for brain activity to steer locomotion	12

## Appendix

### Overview of Scenarios

Scenario 1 (3416). This room of the future revolves around the word “contact,” as we think that residents of the future value being in touch with the world around them and that residents make choices on their own and have some degree of autonomy. The resident has a touch screen to control the room. As the resident will be in bed most of the times, this is an important feature of the room. The bed can be transformed into a chair. The bed is equipped with sensors to monitor the vital functions of the residents, such as heart rate, muscle tension, and blood pressure. The mattress can provide the resident with a massage. There are arm rests to the bed which are foldable and which allow the resident to sit down comfortably. The windows are positioned somewhat lower, so that people can look outside from the bed which is positioned next to the window. There is a seat next to the bed for relatives, so they can be close and make contact. In addition, there is a virtual wall on the opposite side of the bed, stretching across the wall from left to right, projecting images from the past, a holiday movie, or documentary about nature. This enables the resident to be in touch with things from the past as well as the present. In addition, the room is equipped with a virtual ceiling. As the resident is in such a position, lying on his back, looking towards the ceiling, there is actually something to experience on this ceiling. Moreover, there is an “odour box” installed, which emits scents from freshly baked bread, nature, and the sea. All senses should be stimulated, in order to get a complete experience of the world around the resident. In addition to the virtual wall and ceiling, there is the so-called “home wall.” This wall offers space for personal belongings, ranging from books to a fish bowl, from picture frames to a plant or alarm clock. All furniture in the room is mobile and has been programmed in order to meet the needs of the visitors, of professionals, of the resident, as well as a day and night position. The resident has a freedom of choice if he wants to leave the room with his bed and drive towards the common living room. The furniture responds to software which can be operated through the touch screen from the bed and will move aside. There will be a family arrangement of the furniture or a work arrangement. It is also important to have a section in the room allocated to relatives, where they can sit down and work on their laptop as a flexible workplace. The one to one contact can sometimes be replaced by knowing that a relative is near, working in your room.

Scenario 2 (3417). Welcome to nursing home “All-I-Ever-Wanted.” Mrs. Elisabeth Smith is 90 years old and has been living in the nursing home since a couple of months. She has dementia, morbid obesity (weighing 190 kg), diabetes, decubitus, and problems with her blood pressure. She has four children and 12 grandchildren, and five years ago she has lost her husband. They have moved abroad, but via a large screen in her room, she can keep track of their whereabouts and make digital contact with people far away. The room should create an atmosphere of safety and security, as well as homeliness, and with great lighting to create a healing environment. In the morning, the voice of her son living abroad wakes up Mrs. Smith. Whenever her children come to visit, they have to come from afar, and, therefore, a visit needs to be pleasant. Mrs. Smith values atmosphere and hospitality. Therefore, she has a small coffee corner in her room, which can be used by relatives. There are also small stools available next to the bed for children to sit on. Mrs. Smith can control and interact with movement program through moving her feet and hands and touch panels attached to her bed. This enables her to make use of the Internet, listen to the radio, and use the free Wi-Fi facilities in her room, together with a laptop for children. An LED panel behind her back provides feedback to her actions. Mrs. Smith can choose and select/program her own paintings of her liking. She is allowed to consult an interior architect in order to alter her room and change colours of the walls. The indoor climate can also be controlled according to personal preferences. The LED lighting and odours in the room can also be controlled from her bed. Mrs. Smith can alter the atmosphere of the room by changing the colours and shapes of the walls from her bed. A board placed below the bed monitors the vital functions of Mrs. Smith. As an additional feature, the bed has armrests. The bed itself is made of soft material and is positioned in an active posture: looking out over the entrance of the room. Mrs. Smith can turn the bed 360 degrees. This makes Mrs. Smith livelier and makes her more approachable to guests. Moreover, she herself can enjoy all features the room has to offer. She has a number of mobile panels in the room which can be lowered or raised in order to match privacy needs if other people are around. There are also flowers attached to the ceiling for Mrs. Smith to enjoy when she is lying on her bed. The digital era and independence are two key words of the concept.

Scenario 3 (*3418*). Welcome to Peter's “experience room.” Peter is bed-ridden due to muscle problems. The most striking feature is the graciously moving ceiling. All around Peter, materials can change colour and shape. This ceiling can simulate various weather conditions through mild movement (for instance, through the wind), light, and sounds. Moreover, films can be played. Peter is confined to bed day-long, which always makes him be in the same environment. This concept, which can be operated through a Buddy, can give Peter the possibility to change his environment. The ceiling is connected to an interactive wall. This interactive wall has the same function as the ceiling. Through the interactive wall, Peter can make contact with his relatives and with Peter's personal Buddy. The Buddy reminds Peter of important personal things but can also provide counselling in the use of the system itself and other things. Peter's bed is equipped with numerous sensors for, among others, temperature, pressure, skin condition, movement, blood values, and sleep rhythm. Whenever there is something wrong, an alarm is sent to the tablet computer of the carer, who can then take action. There are plants near the bed. In the room, there is a mobile shower, which Peter can page whenever he wants to be showered. The shower turns the bed into a true Jacuzzi. This is enabled through a new innovative material the bed is made of, which can hold water, but which also drains the water with one push on the button. Thereafter, the bed can again be used as a bed. The bed also has various massage functions to alleviate Peter's physical pains. Whenever Peter's partner visits him, the bed can easily be extended into a two-person king-size bed. All medical devices are hidden away in the environment, in order for Peter not to be confronted with the fact he is residing in a nursing home all of the time.

Scenario 4 (*3419*). The resident lies in bed and slowly wakes up. An intelligent system called Service TV in the room recognizes and detects that the resident has woken up and adjusts to the room to the morning situation. The system can be used for communicating with care professionals and a virtual nursing aide. The system activates the sound of birds singing: this helps the resident to slowly wake up. Somewhat later, the system activates the Smart Window, a system which projects dynamic virtual images on an existing window, such as that of a forest with birds flying around and squirrels running from one tree to another. This could also be images from the former neighbourhood, which moves along with the direction of gazing by Mrs. Saunders. The intelligent system seeks contact with the resident when it detects more movement in the morning. “Good morning Mrs. Saunders,” says the system, asking “Did you sleep well?” Mrs. Saunders talks to the smart system, which is a display at the end of the bed and asks the system to switch on the lights. The system asks if Mrs. Saunders wants to finish the movie she was watching the other night or to listen to some music. Mrs. Saunders opts for the music so she can wake up slowly. The system asks if Mrs. Saunders wants to have breakfast and tells, upon request, what is on today's menu. The screen displays the selection. Shortly after, breakfast is served. The bed is moving in an upright position so Mrs. Saunders is in a more convenient position to eat. James, the service robot, serves breakfast. This robot can also be used for transfers of the resident.

Scenario 5 (3406). Once upon a time, there was the nursing home of the future. Mr. Stevens is a resident of a somatic ward. One day, he wakes up and takes his touch screen device. His bed is moving into the upright position and Mr. Stevens opens the curtains. He sees the sunshine outside. The skylight above the bed can also be opened to let in a refreshing breeze. The touch screen can also be used to call a care professional, both by pushing a button or by speech. When the care professional stops by, she rings the doorbell and Mr. Stevens can open the door through his touch panel. He can see through the window who is in front of the door or through the camera of the touch screen. Mr. Stevens needs to get dressed, and his wardrobe is full of clothes. On the touch screen panel, he can browse through his outfits and let the care professional know what he would like to wear. Before getting dressed, Mr. Stevens need to be washed first. Because he is in a two-person king-sized bed, he can automatically move to the side with the help of a conveyor belt inside the bed. There, the care professional has better access to him. Mr. Stevens can also control a mobile wash basin next to his bed, which has sensors to dispense soap and make the water run. If Mr. Stevens prefers to be showered, the conveyor belt can move him to the other side of the mattress and place him onto a mobile shower seat. This seat can take him to the bathroom. Mr. Stevens is about to have visitors today, and their arrival is announced on the touch screen. Through the one-way-mirror-window, he sees a worker from the tax services standing in front. He leaves the door shut. His daughter, however, is most welcome. She brings flowers, which she puts in a vase next to a number of seats, put in place for visitors. Together, they go watch pictures, which his daughter has uploaded online, on a large screen hanging from the ceiling. The care professional joins in. Many functionalities are displayed on this screen, and together, Mr. Stevens and his daughter order a meal. The daughter wants to stay for the night, so the double bed comes in handy. The bed can be split into two separate beds. There are also movement sensors in the bed, also partly to prevent falls, so care professionals are alarmed and can have a look. The bed and mattress have temperature control. The bed frame itself is made of materials that give it a homelike appearance. Some of the technologies below the bed are covered with curtains and wood. The bed is preprogrammed in order to take on certain positions. The mattress itself makes movements to prevent decubitus. All measures contribute to the well-being of Mr. Stevens.

Scenario 6 (3406). “Let's have a look at what time it is. It's time to get up!” Mrs. Johnson is alarmed by her computer screen. She presses the do-not-disturb button. The bed moves into the upright position. With a swaying movement of her arms, Mrs. Johnson can open the curtains and let the sunshine in, as technology recognises the movement and turns it into action. “When I look outside, I have a beautiful view, and hopefully, good weather.” Mrs. Johnson would like a cup of coffee, and a mobile drink machine/minibar pops up next to her bed. She orders breakfast via her tablet computer. On a large display hanging above her, Mrs. Johnson can see the views from the old pub she used to visit, the forest, the street in which the resident used to live, the home of friends, and even the evening news. This allows Mrs. Johnson to stay connected to the daily life of the old environment. In case the resident wants to lie down again, the screen can be raised once more so that it is visible from all corners. The screen itself can be controlled via a tablet computer which is at arm's length and has two sides, one side for functioning care and the other side for leisure and relaxation and leisure, including chatting. If Mrs. Johnson wants to go outside, the bed can be easily changed into a (wheel)chair. The middle piece of the bed can be disconnected, and the side frames remain standing. This allows Mrs. Johnson to ride outside independently. Taking a bath is difficult for Mrs. Johnson as he does not have sufficient strength in his arms. Therefore, bionic arms are put in place. These can be worn by Mrs. Johnson in order to be able to wash herself. In order to offer the best of the best to Mrs. Johnson there is a minibar and a combination oven next to the bed. Visitors can be catered with foods and drinks, which can be served by Mrs. Johnson herself.

Scenario 7 (3403). Anthony is a resident with late-stage dementia. He does not recognize himself and cannot express what he wants and needs. Anthony often gets restless and frightened. Therefore, a sensor has been placed onto the mattress, which registers the changes in behavioural patterns. The mattress is equipped with “pick sensors,” which monitor hand picking movements being made onto the mattress. From analysis from Anthony's personal data, from a period before his late-stage dementia, we know what makes him more relaxed and at ease. One of these things are the personal videos about the family, which are displayed on a large screen, as well as Skype and music, in order to provide a sense of home. Next to the bed, there is a picture frame with digital pictures which shift from one picture to the other, whenever Anthony wants to see pictures of his wife and children. The music is tuned onto the mood of Anthony and the atmosphere. Because it is not desirable to call relatives ten times or more at night, because Anthony may be restless, there is a self-repetitive video image on display, which matches the needs of Anthony. In addition, there are numerous sensors in the mattress which measure vital functions, such as heart rate, blood pressure, muscle tension, respiration, and the amount of medication levels in one's blood. The sensors are self-learning. As you can see, Anthony is getting restless now, and that means his muscle tension is increasing, as well as his heart rate. We then know that he needs some medication to calm down. There are special luminaires mounted to the ceiling above Anthony, for projecting daylight, evening light, and so on. There is also a 3D projector, which has made images of Anthony's former home, living room, and bed room, which are projected onto the wall to make Anthony feel more at ease. There is also an odour dispenser, which emits the scent of Anthony's wife. Sounds of the sea, nature, and the city can be used to further make Anthony feel more at ease. There is also a wind machine to create a nice breeze. With this surround-system, the 3D projector becomes a 4D experience.

Scenario 8 (3403). Mary-Ann has chosen to wake up a little earlier because of television recordings. She put on her best dress, had her hair dressed, and put on her make-up. Of course, she needed help with this because she cannot do all this independently. To Mary-Ann, it is important to be the boss-in-her-own-bed. This means that through the use of a wide range of technological applications, she can control and decide about the things around her. For instance, via a control panel she can decide to open or shut curtains, watch television, and communicate with relatives or friends. Having a sense of autonomy is important to Mary-Ann. Mary-Ann also values the fact that her bed is in fact a “better-bed,” a bed which can be transformed into a chair. “If anyone else can have a seat, why can't Mary-Ann?” This would call for additional adaptations, as Mary-Ann needs assistance with movement and the correction of her body. The new bed enables Mary-Ann to participate in daily activities in a sedentary way. Mary-Ann also cherishes things around her which contribute to the atmosphere, such as lighting, which can be either romantic, or functional for work processes, or suitable for the night so Mary-Ann can go sleep. She can control the lighting herself with the touch screen. In addition, when Mary-Ann is hungry, she wants a proper meal to be delivered. After ordering a meal via the screen, she is in touch with the catering service. When the meal is delivered, she asks the waiter to enter the room. Mary-Ann is also the boss-around-the-bed. Mary-Ann decided who she lets into her room. It is important to create a space in which Mary-Ann feels pleasant. Therefore, a virtual environment is created, in which Mary-Ann can call for functionalities which match her emotions. Although she now lives in a nursing home, she wants to be in touch with her former living room. With one push on a button, her former living room is projected around her, together with her own grandchildren. This makes her feel pleasant and comfortable. She can also bring the outdoor world indoors and see the outdoor weather displayed around her. Pictures, souvenirs, and hobbies can be displayed as well.

Scenario 9 (3403). The bed itself is made for persons with decubitus and is designed in such a way that it can be used to easily turn residents around in bed. This bed is full of sensors which can measure and detect body functions and health status and which should take actions to turn and move residents around the mattress. Moreover, the bed has a function that it can turn from a horizontal stretcher into an upright position, just like a wheelchair so people can easily get out of bed. The bed itself has wheels so it can be moved around. The bed can take action when it is needed for the health of the resident. Mr. Janssen is in this bed. He has a half-sided paralysis due to a stroke, and he has mild dementia. The first modification is related to odours: when these escape from the resident, or when others smell odours (urine, faeces, and wounds) on the corridor, this is considered to be unpleasant. An odour neutraliser should help stop the spread of unwanted odours. An “odour ring” can be placed near wounds to absorb the smell of wounds. There is an interactive ceiling, with projections of Dutch skies full of clouds, which has an effect on the circadian rhythm of the resident, with light being emitted that changes in intensity over the course of the day. Moreover, music and scents are being released by the ceiling. A suspended interactive multimedia screen (music, television, and chatting) is near the foot-end of the bed, which enables the resident to have contact with other rooms in which activities take place. This screen can be controlled via the “bunny toy.” Residents can use this toy-like rabbit to steer the system via voice-control. The resident can thus give spoken commands. The bunny can be used for petting, it is easy to clean, and, in the future, it could be developed for sexual activities in combination with the screen.

Scenario 10 (3408). Mrs. Fontys has turned 50 years old and has been given a balloon flight for her birthday. This flight ended in a personal drama, in which Mrs. Fontys has got an injury high up in the spine. For two weeks, she has been in a rehabilitation ward of a nursing home, where staff is working hard on her recovery. Mrs. Fontys can still move her arms and head, but she no longer has any strength in her hands. The rehabilitation ward has been opened recently, and this offers a number of options. Today, Mrs. Fontys wakes up at 9 o'clock, and, fortunately, she has slept well last night. With the blink of her eyes she calls her Care4You: a large computerised screen. The options provided can be used throughout the day. First, Mrs. Fontys selects care services. On the online time line, she can select a number of times of day during which she receives help, for instance, when breakfast is served, when she is washed, and when she needs to go to the physiotherapist. She receives immediate feedback from the system when care professionals are available to stop by. Because it takes a while for breakfast to be served, Mrs. Fontys chooses on the care4You system to have a cup of coffee served. Bert, the catering robot, stops by and serves coffee via a straw. While enjoying her coffee, she wants to check how her daughter Karin is doing during her test week at school. Via the Care4You system, a connection with communication software is being made.

Scenario 11 (3408). Roderick wakes up and activates his tablet computer. Via a screen, which is projected on the wall, he can be woken up by his grandchild or a care professional. Via another functionality on his tablet, Roderick indicates he wants to go to the toilet. A robot comes to the rescue and is being used to help Roderick go to the toilet. The robot operates a hoist to lift Roderick out of bed and further guides him to the bathroom. The tablet computer can stay somewhere near the bed, as Roderick can control the robot via a Wi-Fi-operated voice control. When Roderick returns from his sanitary break, the robot helps him out of his bed. Roderick is now seated in a chair, ready for something to eat. Roderick takes the tablet computer again and activates a room service app and makes his selection for breakfast. Shortly after, breakfast is being served by a nursing aide. Apart from using technology, face-to-face contact with a care professional, who physically enters the room, remains very important. After his meal, Roderick seeks contact with the outside world. He makes online contact to his daughter's kitchen via the projection screen. This family is also finishing breakfast. Roderick and his family are exchanging the latest news. The projection screen also allows Roderick to seek contact with the common living area of the nursing home, where residents flock together, with whom he can exchange news. The projection screen also serves as a television to watch the news. These news items also serve as input for conversations. In the evening, Roderick is ready to go sleep. The robot is again activated, for instance, to go get a glass of water. Moreover, the robot can detect nocturnal restlessness or help Roderick go to the bathroom again. If Roderick has the strength to try walk around on his own, there is a night orientation light strip on the floor that helps guide him to the bathroom.

Scenario 12 (3408). Barbara cannot function without the help of her tablet computer. She has a critical character and wants to be in control and take care of her own business. Sometimes, she wants to watch television. When pressing her tablet computer, a television screen is lowered from the ceiling. At other times, she wants to go to the park or play cards. Barbara lives in iShell Park II, a cluster of three dwellings which are laid out in a circle, and which can be mutually exchanged for residence. Her personal iShell, a digitalised and mobile environment, is equipped with a large number of facilities: homeliness, a personalised ventilation and temperature control system, a comfortable chair for visitors, sound absorbing surroundings, and a music system, and she can steer around flexibly. These facilities can be controlled via the tablet computer. Her “information glasses” keep her up to date with the latest gossips. The multifunctional iWheel can take her “personal shell” out to the park. This is actually stimulated through the iShell meet and greet, so she can be in touch with others. Via the ShellBook app, Barbara stays in touch with her fellow residents through digital means. The iWheel is multifunctional and is, for instance, equipped with an agenda so she knows when her activities start. Barbara is wearing the so-called iShell cap, which monitors all of her movements and activity. In the future, it is expected that the iShell cap can take over her locomotion and motor skills.
